# A genome-wide association study identifies candidate loci associated to syringomyelia secondary to Chiari-like malformation in Cavalier King Charles Spaniels

**DOI:** 10.1186/s12863-018-0605-z

**Published:** 2018-03-22

**Authors:** Frédéric Ancot, Philippe Lemay, Susan P. Knowler, Karen Kennedy, Sandra Griffiths, Giunio Bruto Cherubini, Jane Sykes, Paul J. J. Mandigers, Guy A. Rouleau, Clare Rusbridge, Zoha Kibar

**Affiliations:** 1 Department of Neurosciences, CHU Sainte Justine Research Center, University of Montréal, 3175 Cote-Sainte-Catherine, Room 3.17.006, Montreal, QC H3T 1C5 Canada; 2 School of Veterinary Medicine, Faculty of Health & Medical Sciences, University of Surrey, Guildford, Surrey GU2 7AL UK; 3 Department of Medical Imaging, London Health Sciences Centre, London, ON N6A 5A5 Canada; 4 Stone Lion Veterinary Hospital, 42 High Street, Wimbledon, SW19 5AU UK; 5 Dick White Referrals, Six Mile Bottom, Cambridgeshire, CB8 0UH UK; 6 Thames Valley Veterinary Services, London, ON N6A 4V2 Canada; 7 Department of Clinical Sciences of Companion Animals, Faculty of Veterinary Medicine, Utrecht University, Utrecht, 3584 CM The Netherlands; 8 Montreal Neurological Institute and McGill University, Montreal, QC H3A 2B4 Canada; 9 Fitzpatrick Referrals, Godalming, Surrey GU7 2QQ UK

**Keywords:** Syringomyelia, Chiari malformation, CKCS dog breed, Cranial MRI measurements, Whole genome association study

## Abstract

**Background:**

Syringomyelia (SM) is a common condition affecting brachycephalic toy breed dogs and is characterized by the development of fluid-filled cavities within the spinal cord. It is often concurrent with a complex developmental malformation of the skull and craniocervical vertebrae called Chiari-like malformation (CM) characterized by a conformational change and overcrowding of the brain and cervical spinal cord particularly at the craniocervical junction. CM and SM have a polygenic mode of inheritance with variable penetrance.

**Results:**

We identified six cranial T1-weighted sagittal MRI measurements that were associated to maximum transverse diameter of the syrinx cavity. Increased syrinx transverse diameter has been correlated previously with increased likelihood of behavioral signs of pain. We next conducted a whole genome association study of these traits in 65 Cavalier King Charles Spaniel (CKCS) dogs (33 controls, 32 with extreme phenotypes). Two loci on CFA22 and CFA26 were found to be significantly associated to two traits associated with a reduced volume and altered orientation of the caudal cranial fossa. Their reconstructed haplotypes defined two associated regions that harbor only two genes: *PCDH17* on CFA22 and *ZWINT* on CFA26. *PCDH17* codes for a cell adhesion molecule expressed specifically in the brain and spinal cord. *ZWINT* plays a role in chromosome segregation and its expression is increased with the onset of neuropathic pain. Targeted genomic sequencing of these regions identified respectively 37 and 339 SNPs with significantly associated *P* values. Genotyping of tagSNPs selected from these 2 candidate loci in an extended cohort of 461 CKCS (187 unaffected, 274 SM affected) identified 2 SNPs on CFA22 that were significantly associated to SM strengthening the candidacy of this locus in SM development.

**Conclusions:**

We identified 2 loci on CFA22 and CFA26 that contained only 2 genes, *PCDH17* and *ZWINT*, significantly associated to two traits associated with syrinx transverse diameter. The locus on CFA22 was significantly associated to SM secondary to CM in the CKCS dog breed strengthening its candidacy for this disease. This study will provide an entry point for identification of the genetic factors predisposing to this condition and its underlying pathogenic mechanisms.

**Electronic supplementary material:**

The online version of this article (10.1186/s12863-018-0605-z) contains supplementary material, which is available to authorized users.

## Background

Canine syringomyelia (SM) is a painful condition where fluid-containing cavities (syrinx or syringes) develop within the parenchyma of the spinal cord and which progress over time [1, 2]. Depending on the site of spinal cord damage, SM may result in behavioral signs of pain, fictive scratching, scoliosis, weakness and sensory deficits [3]. Approximatively 70% of older Cavalier King Charles spaniels (CKCS) have MRI signs of SM. This high percentage seems to correlate with another condition present ubiquitously in this breed called Chiari-like malformation (CM) [2]. CM is a complex developmental malformation of the skull and cranial cervical vertebrae that is characterized by rostro-caudal bony insufficiency resulting in conformational changes and overcrowding of the brain and cervical spinal cord particularly at the craniocervical junction. Obstruction of the foramen magnum and cerebrospinal fluid (CSF) channels is hypothesized to be pivotal in the pathogenesis of SM [4–7].

In the CKCS, risk of SM has been shown to be associated with increased brachycephaly with rostrocaudal doming i.e. a heightened cranium that slopes caudally [8] and reduced skull base due to craniosynostosis or premature skull suture closure [9]. The underlying pathogenic mechanisms proposed for the development and progression of SM secondary to CM remain poorly understood and even controversial complicating the interpretation of many clinical observations and the selection of the appropriate treatment protocols. Current prevailing hydrodynamic theories generally assume that syrinx fluid is CSF that has entered the cord as a result of perturbations of pulsations in the subarachnoid space caused by overcrowding of neural parenchyma in the caudal part of the cranial fossa and the cervical vertebral canal as seen in CM [10].

Studies on the inheritance of SM have shown that it is a complex trait with a moderately high heritability [11]. The genetic origin of CM and its role in SM remain to be identified. Incomplete penetrance and variability of clinical signs in both CM and SM seem to indicate a polygenic mode of inheritance [11, 12]. The genetic approach widely used to investigate complex disorders is a genome-wide association study (GWAS) that aims at identifying genes or SNPs that determine the disease even though each gene contributes only a small fraction to the disease process. This strategy was applied successfully in dogs and permitted the identification of loci associated with osteoarthritis of hip joints [13], hip and elbow dysplasia [14], and related *BMP3* (*BONE MORPHOGENETIC PROTEIN 3*) variations to skull diversity [15]. Particularly, an association study was successfully used to identify loci associated with CM in the Griffon Bruxellois (GB) breed. A total of 14 quantitative skull and atlas measurements were taken and were tested for association to CM in the GB. Significant associations were identified between specific traits and CM/SM status in GB [16, 17], a mixed GB breed [18] and CKCS cohorts [6]. A GWAS in the GB cohort identified one locus on CFA2 (CFA, *Canis Familiaris* autosome) strongly associated to the height of the cranial fossa and another locus on CFA14 associated to both the height of the caudal cranial fossa (reduced supraoccipital bone) and brachycephaly [17]. These two loci were significantly associated to CM further strengthening their candidacy. In this study, we conducted a GWAS to identify genomic regions that predispose to SM secondary to CM in the CKCS dog breed.

## Methods

### Cohort and phenotypic traits

A cohort of 96 CM affected CKCS with DNA consisting of 40 males (26 affected and 14 unaffected) and 56 females (35 affected and 21 unaffected) with an average age of 5.5 ± 2.5 years was included in the quantitative study investigating CM (Additional file 1: Table S1). This cohort was part of a larger CKCS cohort used by the same group to characterize painful CM and secondary SM for which DNA was available [6]. DICOM (Digital Imaging and Communications in Medicine) T1-weighted midsagittal MRI of the brain and cervical region of these 96 dogs allocated with an ID were analyzed. The minimum inclusion criterion was visualization of the hindbrain to the level of the interthalamic adhesion to the cervical vertebrae 4/5 intervertebral disc space. Only DICOM images accompanied by details of birth, date of MRI and identity microchip number were used. The MRI studies were loaded into DICOM viewing software eFILM workstation (Merge Healthcare 900 Walnut Ridge Drive, Hartland, WI 53029 USA). CM/SM status was determined by author CR by noting the presence of CM and SM on sagittal T1 and T2W weighted images and then, if SM was present, determining the maximum internal syrinx transverse diameter (STD) from transverse T1 weighted images of the cervical spinal cord. The smallest unit of measurement in eFILM is 1 mm (mm). These criteria are in accordance with the British Veterinary Association/ Kennel Club guidelines for screening for SM (https://www.bva.co.uk/Canine-Health-Schemes/CM-SM-Scheme/). SM severity was established according to the STD as wide STD has been previously associated with clinical signs of pain, fictive scratch and scoliosis [19]. The 96 CKCSs were separated in 3 categories that took account of late onset condition of SM: normal (35 dogs without syrinx or central canal dilation (STD = 0 mm over 5 years of age), intermediate (29 dogs with STD = 1 or 2 mm over 5 years of age) or severe (32 dogs with STD ≥ 3 mm any age).

**Fig. 1:**
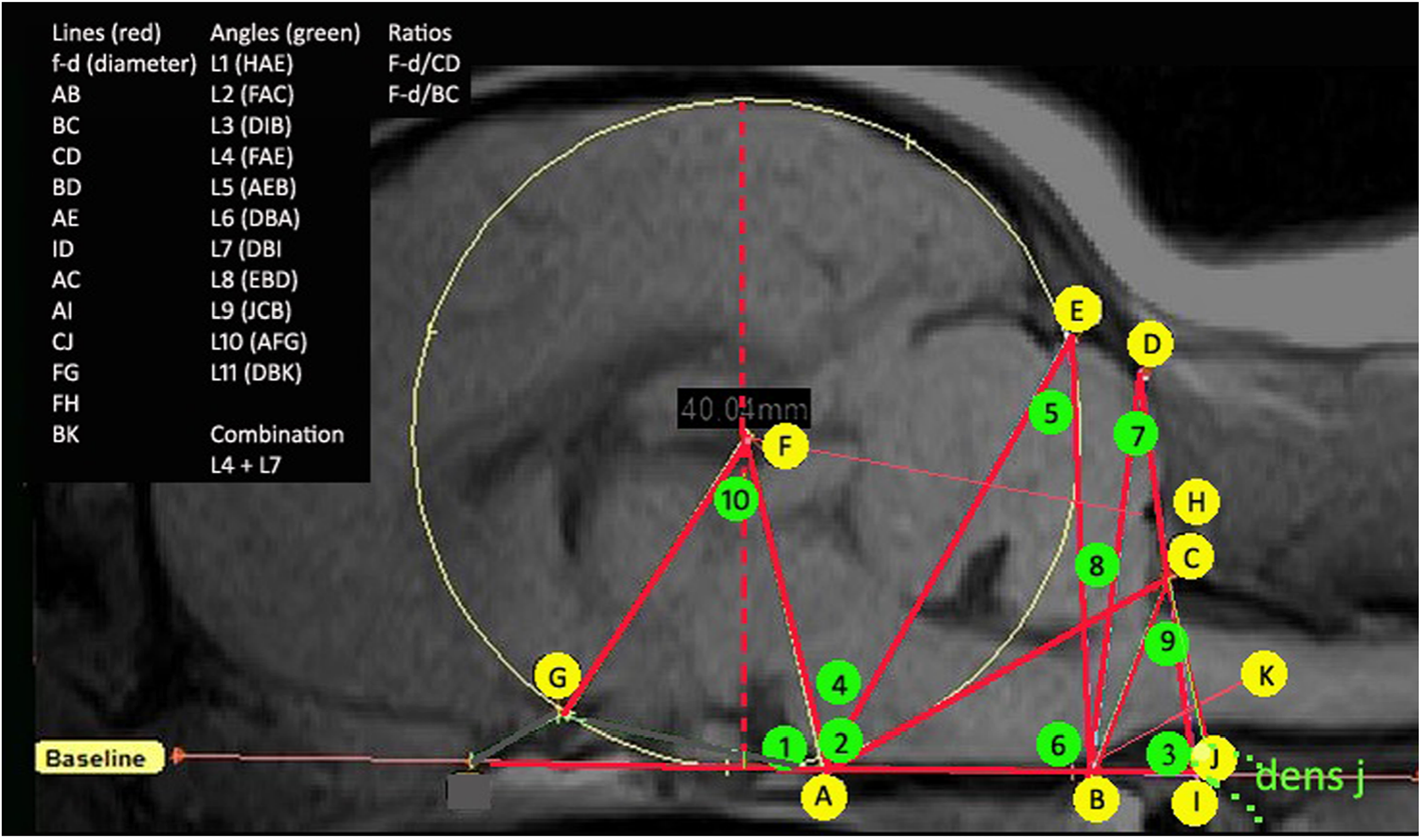
Morphometric measurements of a Cavalier King Charles Spaniels whole brain. Measurements were chosen to best reflect the possible morphological changes observed in SM. All measurements start from one of these points: **a** dorsum of sphenoid-occipital synchondrosis, **b** basion of basioccipital bone, **c** rostral edge of the dorsal lamina of the atlas, **d** junction between supraoccipital bone and occipital crest, **e** most dorsal point of intersection of the cerebellum with the occipital lobe circle, **f** center of occipital lobe circle, **g** point at which the optic nerve deviates into the optic canal, **h** rostral edge of supra-occipital bone, **i** intersection point with the extended AB baseline caudally with extended line DC ventrally, **j** most rostral aspect of the dens of the axis bone and **k** extended line from point B along the best fit line of the ventral medulla oblongata to where it changes angle to the spinal cord

**Fig. 2:**
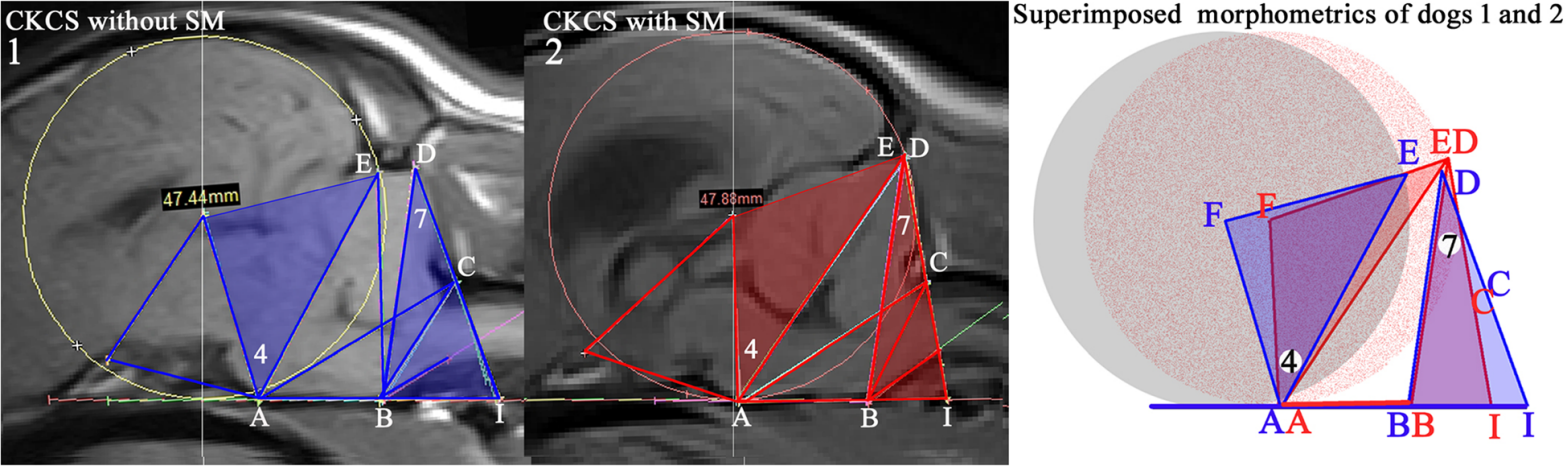
Cavalier King Charles Spaniel with and without SM illustrating differences in the size and arrangement of a combination of angles 4 and 7 (*L*4 + *L*7). Angles 4 and 7 are smaller in the CKCS with SM (red) as compared to CKCS without SM (blue) reflecting a reduced cranial caudal fossa and rearrangement of neural parenchyma. Right panel, a schematic Fig. of the occipital circle with centre F (grey) and angles 4 + 7 of the CKCS with SM (red) that have been superimposed on the CKCS without SM (blue). A, dorsum of sphenoid- occipital synchondrosis; B, basion of basioccipital bone; D, junction between supraoccipital bone and occipital crest; E, most dorsal point of intersection of the cerebellum with the occipital lobe circle; F, center of occipital lobe circle

Next, using a DICOM reading software (Mimics® 14.12, Materialise, Belgium), a total of 11 structures were defined and 27 lines, angles and ratios were measured by SPK, initially blinded to SM status (Fig. 1, Additional file 1: Table S1). The mapping of the hindbrain and craniocervical junction was adapted from previous genetic and conformational studies undertaken in the Griffon Bruxellois [16, 17]. Dogs with narrow syringes or small central canal dilation are more likely to be asymptomatic at least with regard to the syrinx. The CKCS breed is very variable in size and head-shape. In view of this, two ratios were taken which reflected the size and shape of the caudal fossa and relating this to the height of the cranial fossa (f-diameter). The first ratio was the f-diameter (F-d) divided by the distance across the foramen magnum from the caudal point of the basioccipital to the rostral point of the atlas (line BC) and the second was F-d divided by the height of the supraoccipital bone (line CD) (Fig. 1). Since the MRI of the forebrain and olfactory bulb was not always available for analysis, in order to take account of the impact of brachycephaly and compensatory rearrangement of parenchyma and reduced caudal fossa, the two angles which most reflected these deviations in the hindbrain, angle 4 and angle 7 were combined (*L*4 + *L*7) (Fig. 2). The hypothesis being the smaller these two angles were, the greater the deviation and reduction in the neural parenchyma of the hindbrain and craniocervical junction.

### Genotyping across the genome

A total of 96 CKCSs were genotyped with CanineHD Genotyping Beadchip (Illumina Inc., San Diego, CA, USA) which contained 173,662 SNPs. These SNPs were analysed using genome studio and filtered for SNPs with a minor allele frequency > 0.05, a SNP genotyping rate of 0.9 and an individual genotyping rate of 0.9 using Plink V1.07. A total of 85,647 SNPs were excluded for lack of informativeness in the CKCS breed (MAF < 0.05). Three dogs had a genotyping rate under 0.9 and were therefore excluded from further analysis resulting in 93 dogs (Initial cohort: 39 affected, 54 unaffected, 39 males, 54 females). To remove potential bias associated with ambiguous phenotypes, dogs with STD less than 3 mm were removed from the analysis. This resulted in a final cohort of 65 dogs (32 affected, 33 unaffected, 26 males, 39 females) genotyped with 88,015 SNPs.

### Targeted next generation sequencing

The 2 loci of 0.17 Mb and 0.8 Mb on CFA22 and CFA26 associated to ratio F-diameter (F-d)/BC and *L*4 + *L*7 respectively were sequenced in the same 65 dogs used for GWAS using SeqCap Custom relaxed probe set library preparation specifically designed by Roche Nimblegen (Madison, WI, USA) and subsequently subjected to 100 base pair, paired end sequencing on the Illumina HiSeq2000 Platform at the McGill University and Génome Québec Innovation Centre. Using an SSAHA algorithm, the baits contained 3313 probes with up to 20 close matches in the genome for the purposes of providing maximum coverage. The vast majority of the probes were unique, with a few probes that had a greater degree of multi-locus homology to increase coverage in all regions. Reads were mapped to the genome (CanFam3.1 release September 2011) with Burrows-Wheeler Aligner (BWA). Duplicated reads were removed, the rest were locally realigned using GATK v2.6.4 and annotated using Annovar. The average read depth for the targeted regions was 151,01X (89,32X - 208,32X) with 99,58% of targeted regions covered at greater than 20X.

### Genotyping with tagSNPs

Tagging SNPs were selected to maximize coverage of each locus. They were identified using the tagger routine in the Haploview V4.2 software with a maximum r^2^ of 0.2. Two tagSNPs were amplified on CFA22 at position 13,804,718 and 13,933,606 (rs23040347) with the probes GGATTACAGAAGTCACAGTCGAAAGACTGGGAAAGAGACACCAGAGCTCCAAGTTTATAAAGTTGTATTTTAAAGATTCAGTGATGTCTGAGGAATGAAATGGGATGAGGAAGGAAAAATTATGCACTAGGAGCAATGTTTTCTGTCTTC**[T/A]**GGAATGGGAAGTGAGATGAACAGCATAGGAGATTTGAAAGGTAGCAAACAATCCAGGAGACCTACAGGCCCGTGATCAATGACTTATAAGATGATATTAAGGAAACATTATATATGATACTATATCCTCCTTTGAGAGTCTGTATGCATT and TTAATCTTCAAAACGGCCCAATGAGGATATCCCCATTTTGCAGATGAAGAATGAAGGAACGTTGAAGTTCAGTGATTTGATCCAGAATAAGTGCAGTGAGACTTCAGATCCAGCTATGGGGTTTGCCAAATTCAATCTCTGCCCTTCCTC**[A/C]**CTATTCTTAACCGCCAAATATTATTTATATTTGTAAGAATGCAGTTTTAAAGGTTGAAATTTTCAACTTCTCACACAGAGCAGATAGCTGGAGACGAAGATGGTAGGACTGCTCTCTCATTGCCTGCATTGTGCTCTCTGAGTAGTGAAA respectively. Two SNPs were also amplified on CFA26 at position 32,757,080 (rs23302138) and 32,797,595 with the probes TGTCCTCCTGGCTTCTGAGGGGGTGGGTGCGGGGCCTGGAGGCCCAGAGGGGAACAGGATGTGGCCACAGGATGGAGAGCTGACTTGTGCACAGGGGCCTGTGTGGGTCAGTCTGTGTCCCCGGCACCCCTGAAGCTGCAGGTGTCTCAG**[T/C]**AGAGCCCCTCAGTGGGTAACTCTGCCCCCAATTCCCTCCTTGGAGACTGCATCTCCTCCTGTGCCTCCTGCAAGTCGCTGTCAGCTTCCCTCCCCTGAGGTCTGACGCCTCCTGCAGGAAGTTCTCTGGGATTGGATCTCAAAATGGTGC and CATGAGTTGGAAGGGCAGTTAAGGGCAGAAGGACTTAGAGGCGGAGAGCATAGAGAAGGAAAAGGCACGATGGTGTGTTTGATTATCTCCCCCTCTCCATTCTCATGGTGCCACCTATCCTAATTCCAGTTCGTATTATCATAGGTCTCA**[C/T]**TCCACCAATAACGTCTCAATCACACACACCATGTCCTGTCTTCTCGTTGGTCTGTGCTATGATCTGTGTGGTTCTTCTCTTTCCCAGGAGGCCAGATCTGTATTTTGCTGATTACAATCTACTCTTTAATTCTGGATTGAATTGCTAACT respectively. Genotyping was performed using the TaqMan assay (Applied Biosystems) on 393 CKCS dogs (187 SM unaffected, 274 SM affected).

### Statistical analyses

Initial GWAS analysis was done in 2 phases: association of the quantitative traits (skull and cranial cervical measurements) to disease, followed by association of SNPs to these quantitative traits. Association of the quantitative traits to the disease was done using a linear regression with a type III sum of squares in R V3.0.1 [20] and age as a covariate. Due to the strong association of STD size and age, inclusion of age as a covariate was used to correct for its potential confounding effect. Association of SNPs to the quantitative traits was done using a mixed linear model including age as a covariate and potential stratification as a random effect which was corrected using a genomic relationship matrix using GEMMA V0.94 [21]. All *P* values obtained from the association were corrected together for multiple testing using a storey’s q value method [22]. Haplotypes surrounding these SNPs were reconstructed using Haploview V4.2 [23] and associations with the initial quantitative trait were run using Plink V1.07 linear routine [24] with age as a covariate. Correction was applied using 10,000 permutations. Plink V1.07 logistic regression routine using age as a covariate was used to test for association between tag SNPs and SM and Bonferroni was used to correct for multiple testing (0.05/4 = 0.0125).

## Results

### Association of skull measurements to SM in the CKCS breed

**Fig. 3:**
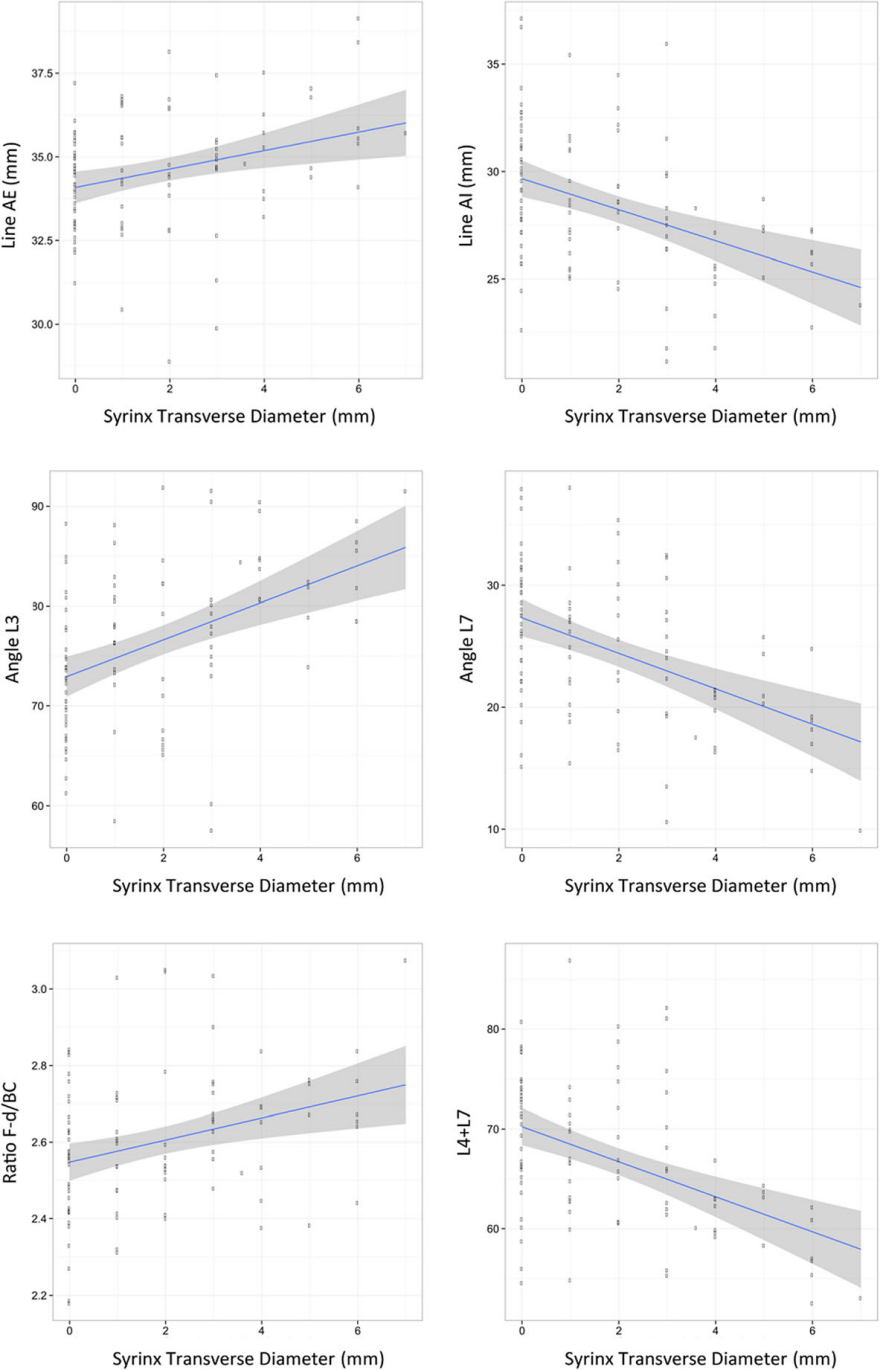
Linear regression of traits and STD size including age as a covariate. A total of 6 measurements consisting of line AE, line AI, angle 3, angle 7, ratio F-d/BC and *L*4 + *L*7 all showed a significant association to STD size (P_bonferroni_ < 0.0019)

The complex skull morphology of the CKCS breed was investigated using 27 lines, angles and ratios as well as age, gender and ventricular dilatation on a cohort of 96 dogs (Figs. 1 and 2, Additional file 1: Table S1). Level of affliction of dogs was defined by the STD size. A linear regression including age as a covariate was used to associate traits and STD size. STD size did not show any association to gender (Table 1). A total of 6 measurements consisting of line AE, line AI, angle 3, angle 7, ratio F-d/BC and *L*4 + *L*7 all showed a significant association to STD size (P_bonferroni_ < 0.0019) and were therefore further investigated (Fig. 3 and Table 1).

**Table 1:** Quantitative traits that are significantly associated (*P* value< 0.05) to syrinx transverse diameter following linear regression and multiple testing correction

Trait	Raw *P* value	Bonferroni corrected *P* value*
Gender	0.680431	1
Age	8.01E-06	0.000216
F-Diameter	0.005901	0.159332
Line AB	0.609389	1
Line BC	0.172273	1
Line CD	0.815288	1
Line BD	0.068142	1
Line AE	0.00124	**0.033479**
Line ID	0.17867	1
Line AC	0.084552	1
Line Ai	7.82E-05	**0.002113**
Line CJ	0.014438	0.389822
Line FH	0.952342	1
Line FG	0.151877	1
Line BK	0.03617	0.976588
Angle *L*1 (hae)	0.769033	1
Angle *L*2 (fac)	0.038195	1
Angle *L*3 (dib)	8.11E-06	**0.000219**
Angle *L*4 (fae)	0.124912	1
Angle *L*5 (aeb)	0.329471	1
Angle *L*6 (dba)	0.278602	1
Angle *L*7 (bdi)	1.01E-05	**0.000272**
Angle *L8* (ebd)	0.861691	1
Angle *L*9 (jcb)	0.136343	1
Angle *L*10 (afg)	0.036817	0.994072
Angle *L*11 (ebk)	0.100105	1
Ratio F-d/CD	0.473593	1
Ratio F-d/BC	0.00172	**0.046441**
*L*4 + *L*7	1.33E-05	**0.000358**

*P values in bold represent significant association

### Genome-wide association study of SM in CKCS

**Fig. 4:**
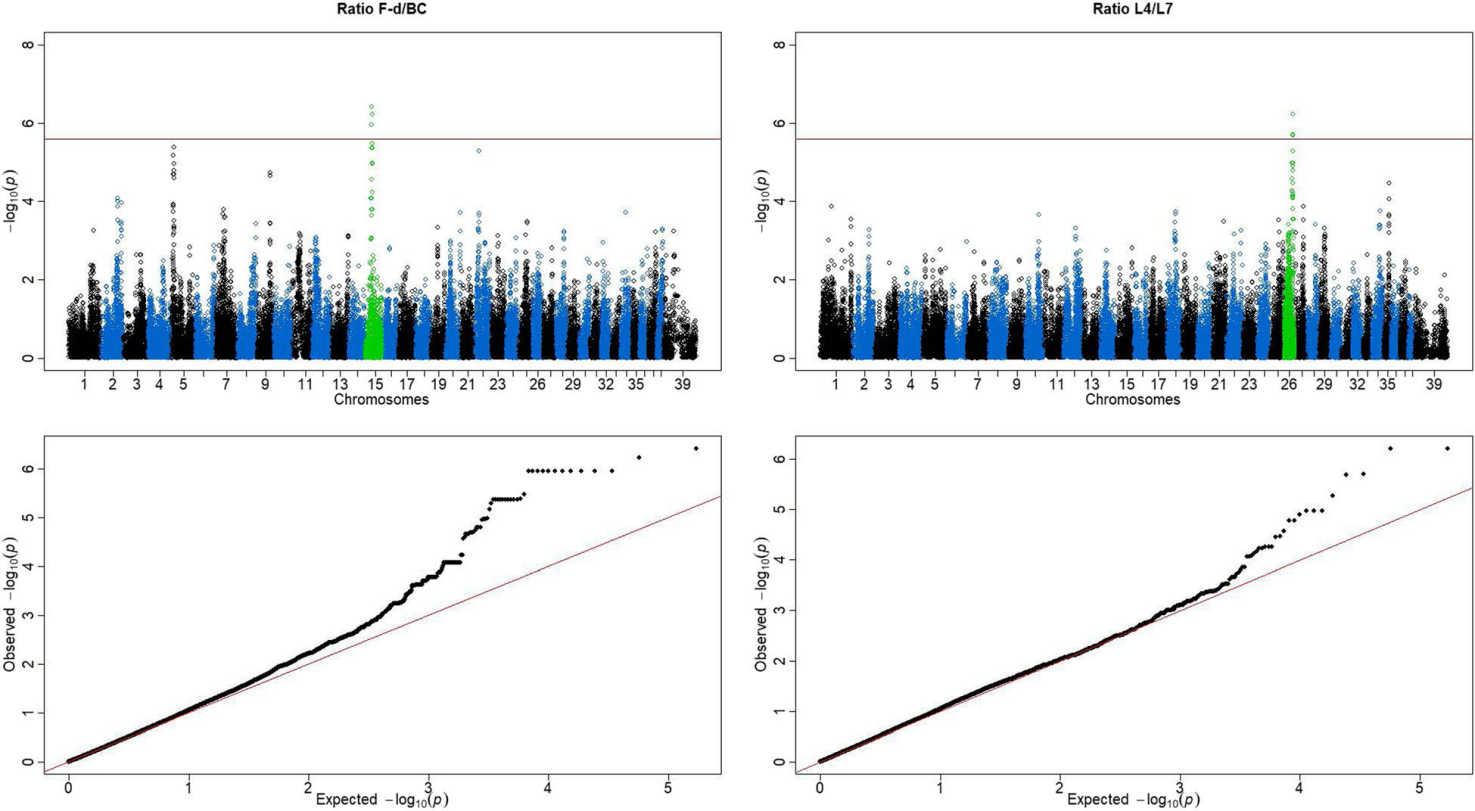
Manhattan plots (top) and QQ plots (bottom) of significant loci obtained by a mixed linear model in traits: ratio F-d/BC (left) and *L*4 + *L*7 (right). Two loci on CFA15 and CFA26 were significantly associated to ratio F-d/BC and *L*4 + *L*7 respectively

A GWAS using a mixed linear model with age as a covariate was applied on the previously identified traits (Line AE, line AI, angle 3, angle 7, ratio F-d/BC and *L*4 + *L*7). This resulted in the identification of a group of 13 SNPs on CFA15 (BICF2S23761321, BICF2G630435380, BICF2S22961368, BICF2G630437186, BICF2G630437178, BICF2G630437135, BICF2G630437112, BICF2G630437075, BICF2G630437073, BICF2S23311892, BICF2G630437043, BICF2G630437038, BICF2G630437002) associated to ratio F-d/BC under a FDR of 0.05 (all *P* = 0.03754) and two SNPs on CFA26 (BICF2P174010, BICF2P152116), which were significantly associated to *L4 + L7* under a FDR of 0.05 (both *P* = 0.03754) (Fig. 4 and Table 2).

**Table 2:** Loci significantly associated to ratio F-d/BC and L4 + L7 in the mixed linear model

Chr	SNP	Position	Raw *P* value	FDR corrected *P* value
15	BICF2S23761321	28,798,671	3.81E-07	0.037545
15	BICF2G630435380	29,147,043	5.87E-07	0.037545
15	BICF2S22961368	26,586,223	1.11E-06	0.037545
15	BICF2G630437186	26,599,059	1.11E-06	0.037545
15	BICF2G630437178	26,605,637	1.11E-06	0.037545
15	BICF2G630437135	26,619,845	1.11E-06	0.037545
15	BICF2G630437112	26,623,178	1.11E-06	0.037545
15	BICF2G630437075	26,645,302	1.11E-06	0.037545
15	BICF2G630437073	26,645,969	1.11E-06	0.037545
15	BICF2S23311892	26,690,382	1.11E-06	0.037545
15	BICF2G630437043	26,734,763	1.11E-06	0.037545
15	BICF2G630437038	26,738,248	1.11E-06	0.037545
15	BICF2G630437002	26,797,343	1.11E-06	0.037545
26	BICF2P174010	32,735,128	5.99E-07	0.037545
26	BICF2P152116	32,738,238	5.99E-07	0.037545

Multiple SNPs were suggestive of association with FDR corrected scores between 0.05 and 0.1 (Additional file 2: Table S2). By comparing the list of these “borderline” SNPs to a previous association study of CM in the GB breed conducted by our group [17], we were able to identify an overlap with only one SNP on CFA22, BICF2P1045632, that was associated with ratio F-d/BC with a *P* value after FDR of 0.07846 in the present study. This SNP maps to a region that was identified as suggestive of association with the line BC in the previously studied GB breed [17]. A replication between two unrelated cohorts from two different breeds offers strong evidence for association strengthening the candidacy of this SNP and suggesting the need for inclusion in the following genetic studies. The other SNPs that did not reach statistical significance in this study and that were not replicated in our previous association study of CM in GB dogs require further genetic analyses in larger cohorts.

### Haplotype analysis of the candidate SM loci

**Fig. 5:**
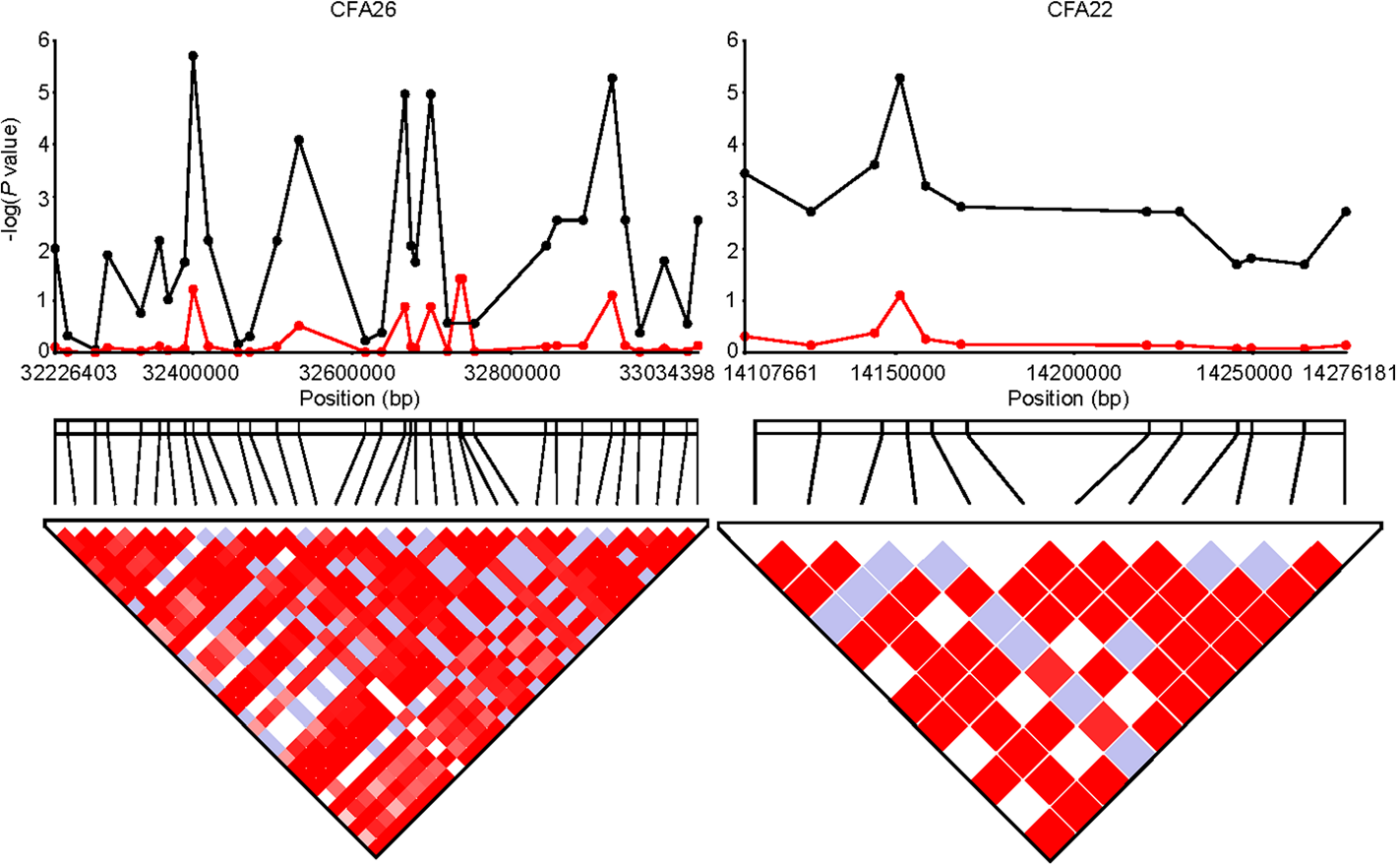
*P* value distribution inside the CFA26 (32226403-33034398 bp) and the CFA22 (14107661-14276181 bp) candidate loci. Top graphs represent the *P* values before correction (black line) and after FDR correction (red line) for the SNPs in reconstructed regions

Using Haploview V4.2, blocks of linkage disequilibrium (LD) surrounding the SNPs significantly associated to *L*4 + *L*7and ratio F-d/BC were defined. A first LD block of 1.7 Mb surrounding SNPs associated to ratio F-d/BC was identified on CFA15 from 24,537,882 bp to 26,252,411 bp (Additional file 3: Fig. S1). A second LD block of 0.8 Mb surrounding SNPs associated to *L*4 + *L*7 was identified on CFA26 from position 32,226,403 bp to 33,034,398 bp. A last LD block of 0.17 Mb surrounding the SNP associated with ratio F-d/BC and identified previously in the GB cohort was identified on CFA22 from 14,107,661 bp to 14,276,181 bp. The three haplotypes were analysed using linear regression that included age as a covariate and looked at the potential association of these blocks with their respective traits. Linear regressions are known to be sensitive to rare haplotypes associated to extreme measurements; therefore, rare haplotypes with a frequency under 0.05 were excluded. This resulted in the identification of three haplotypes, one on CFA22 (*P* = 0.009599) and two on CFA26 (*P* = 0.01067 and 0.00231), that were significantly associated to ratio F-d/BC (Table 3) and *L4 + L7* (Table 4) respectively (Fig. 5). The strong association of these haplotypes and SNPs with their respective traits combined with the strong association of these traits with the STD size support the implication of these regions in the development of SM. No significantly associated haplotypes were identified on CFA15 excluding his region from further analyses.

**Table 3:** Raw and permutation P values of the F-d/BC-associated haplotypes in the CFA22 candidate region at 14107661-14276181 bp

Name	Haplotype	Frequency	Raw *P* value	Corrected *P* value
**1**	AGCCGTCCCTTG	0.431	0.956	1
**2**	GAACATTGCGTA	0.351	0.262	0.7261
**3**	AGCTGGCCTTCG	0.100	0.00153	0.009599
**4**	GGACAGCCTTCG	0.041	0.0644	0.2626
**5**	GAACATTGCTTA	0.062	0.223	0.659

**Table 4:** Raw and permutation P values of the F-d/BC-associated haplotypes in the CFA26 candidate region at 32226403-33034398 bp

Name	Haplotype	Frequency	Raw *P* value	Corrected *P* value
**1**	TTCCCGACAGACGGTAATTGTGTTATAATGTTA	0.215	0.00237	0.01067
**2**	CCGTAAGTAAGAAGGCAGCGCGTCTCGAAACCG	0.492	0.903	1
**3**	TTCCAAGTAAGAAGGCAGCGCGTCTCGAAACCG	0.0538	0.562	0.998
**4**	TTCCCGACAGACGGTAATTGTGTTTCGAAACCG	0.0308	0.434	0.9865
**5**	CTCTAAGTGAACAATAGGCATACTTCGGAGCTG	0.108	0.000584	0.00231
**6**	CTGTCAATGAACAGTAGGCATACTTCGGAGCTG	0.0231	0.0584	0.3657
**7**	CCGTAAGTAAGCAATAGGCATACTTCGGAGCTG	0.0154	0.724	1
**8**	TTCCCGACAGACGGTAATTGTACTTCGGAGCTG	0.0154	0.301	0.9372

### Targeted next generation sequencing of the SM-associated loci on CFA22 and CFA26

Each of the SM- associated regions harbored only one coding gene: *PCDH17* (*PROTO-CADHERIN 17*) on CFA22 and *ZWINT* (*ZW10 INTERACTING KINETOCHORE PROTEIN*) on CFA26. In order to identify potential SM -predisposing mutations, both CFA22 and CFA26 candidate regions were submitted to targeted next generation sequencing. The reads of the sequences obtained from the 65 CKCS were aligned on CanFam 3.1 resulting in 5608 SNPs on CFA22 and 10,814 SNPs on CFA26. Except for one SNP (rs2305483), that was identified in the coding region of *PCDH17* and that was non-conserved and synonymous, all identified SNPs resided in intergenic and deep intronic regions. Using improved coverage of the region, linkage disequilibrium blocks were reevaluated based on the significant SNPs in the regions. A total of 37 and 339 SNPs were defined as significantly associated to F-d/BC and *L4 + L7* respectively. Based on the hypothesis that causative SNPs would be significantly associated to their respective trait, these SNPs refined the regions of interest to 13,785,828-14,183,295 bp (397,467 bp) on CFA22 and 32,721,057 bp to 33,094,292 bp (373,235 bp) on CFA26.

### Genotyping of an extended CKCS cohort with SNPs significantly associated to ratio F-d/BC and *L4 + L7*

To investigate the potential association between the identified SNPs and SM, a cohort of 461 CKCS including 187 unaffected and 274 SM-affected (that included the original 65 dogs) were genotyped with two TagSNPs from each of the associated regions on CFA22 and CFA26. While the 2 selected TagSNPs on CFA26 at position 32,797,595 bp and 32,757,080 bp did not show any significant association to SM (*P* value = 0.7637 and 0.7614), the 2 selected TagSNPs on CFA22 at position 13,933,606 bp and 13,804,718 bp reached significance (*P* value = 0.0104 and 0.02309). Bonferroni corrected P value of the TagSNP at position 13,933,606 bp on CFA22, was still significant at a P value of 0.0104. Hence, we successfully identified a region on CFA22 associated to ratio F-d/BC and SM in the CKCS dogs.

## Discussion

Studies of CM in large affected CKCS pedigrees suggested a polygenic inheritance with a wide clinical spectrum where CM with SM represents the most aggravated form [11, 25]. The genetic factors predisposing to CM and SM have been shown to be interrelated and seem to have their origin in bone development with hypoplasia of the supra and basisoccipital bones and reduced caudal fossa volume associated with a compensatory increase in height of the cranial fossa [7]. Other associated abnormalities include: reduced occipital crest; rostral displacement of the atlas and axis (atlantooccipital overlapping); medulla oblongata elevation/kinking; more acute angulation of the axis bone to the cranial bases (cervical flexure); more acute angle at the spheno-occipital synchondrosis (sphenoid flexure) [4, 6, 7, 26, 27]; reduced volume of jugular foramen and venous sinus [28, 29]; a relatively large cerebellum [10, 30, 31], and dorsal compression from atlantoaxial bands [32, 33]. While some of these traits may be a consequence or insufficiency of the occipital sclerotomes (paraxial mesoderm) which form the skull base and parts of the atlas and axis and associated ligaments [1], we cannot exclude a complex origin where multiple genes lead to a range of phenotypes regrouped as CM with SM.

As previously demonstrated, cranial morphometric measurements can provide significant information to decompose the complex nature of CM and SM [6, 16, 17]. In this study, we identified 6 traits (line AE, line AI, angle 3, angle 7, ratio F-d/BC and *L4 + L7*) significantly associated to STD size. Genetic investigation of these traits identified significant association with SNPs on CFA15 with F-d/BC and on CFA26 with *L4 + L7*. Both these traits represented a combination that demonstrated a reduction in the overall size of the caudal cranial fossa and rearrangement of the neural parenchyma (Fig. 2). Screening of SNPs that were suggestive of association identified one SNP on CFA22, BICF2P1045632, associated with F-d/BC, that mapped to a region found to be associated with the line BC in a previous association study of CM in the GB breed [17]. This replication strengthens the candidacy of the locus on CFA22 for further genetic investigation. Haplotype analysis of candidate loci on CFA15, CFA26 and CFA22 identified significantly associated haplotypes only on CFA22 and CFA26 loci, but none on the CFA15 locus. This suggests that CFA15 initial scores were driven by a rare haplotype overrepresented in extreme cases. Line BC measures the distance from the caudal end of the basiocciput to the atlas, across the foramen magnum. Previous association of line BC to CM in GB strengthens the candidacy of F-d/BC as an important factor in CM and SM etiology [16, 17]. *L4 + L7* was found to be smaller in SM dogs as compared to no SM dogs in linear regression analyses of SM-significantly associated traits. This suggests that the reduced caudal cranial fossa representing CM has a direct influence on SM. Replication of these findings in a larger cohort could provide better diagnostic tools of SM in CM-affected dogs.

Genotyping of tagSNPs selected from both candidate loci on CFA22 and CFA26 in an extended cohort of 461 CKCS (187 SM unaffected, 274 SM-affected) identified a significant association of the candidate locus on CFA22 and SM. Hence, this candidate locus that was suggestive of association to the ratio F-d/BC in CKCS and to the line BC in a previous association study in GB dogs was found to be significantly associated to SM, strengthening its candidacy for SM. On the other hand, the tagSNPs on CFA26 that was significantly associated to *L4 + L7* were not significantly associated to SM in the larger cohort. In a parallel study which characterized the phenotype of CM in the CKCS, two different skull conformation anomalies were identified which resulted in SM in this breed [6]. The trait of smaller *L4 + L7* was a feature of one conformation anomaly (case 2, Fig. 5 in reference [6]). It is therefore possible that significance of this trait in the larger CKCS cohort was reduced since other associated features were underrepresented. By contrast, higher F-d/BC increased risk of SM in both CKCS skull anomalies.

The associated loci on CFA22 and CFA26 harboured each only one gene: *PCDH17* and *ZWINT* (respectively). Targeted next generation sequencing of both CFA22 and CFA26 candidate loci identified a total of 37 and 339 significantly associated SNPs with ratio F-d/BC and *L4 + L7* respectively. No mutation in the coding region of either gene was detected, except for one synonymous mutation in *PCDH17*. We hypothesize that predisposing mutations in these two regions are most likely regulatory that would affect RNA expression of either *PCDH17* or *ZWINT* or other unannotated transcripts. Alternatively, these regulatory mutations could have a long-range expression effect on transcripts residing outside the 2 candidate regions. RNA-sequencing or quantitative RT-PCR studies in affected tissues from dogs carrying the associated haplotypes are needed to test this hypothesis.

*PCDH17* (*PROTOCADHERIN 17*) belongs to the family of protocadherins that are involved in the adhesion and sorting of cells during tissue morphogenesis. It is expressed specifically in several regions of the developing and adult brain and spinal cord [34–41]. It regulates spine development, presynaptic assembly, vesicle accumulation and transmission in corticobasal ganglia synapses [34]. Overexpression of *PCDH17* in primary cortical neurons is associated with significantly decreased dendritic spine density and abnormal dendritic morphology [42] and it is possible that variants in the dog gene could play a role in development of the neural tissue and affect CM/SM disease expressivity. Additional knockout studies in cell or animal models are needed to further investigate the potential role of this gene in the pathogenesis of SM. *ZWINT* (*ZW10-INTERACTOR*) has an important role in kinetochore assembly and proper chromosome segregation [43]. In rats, it was shown to be expressed in different regions of the brain and in dorsal horn laminae and its expression levels increased with the onset of neuropathic pain after chronic constriction injury of the sciatic nerve [44–46]. We tested for association between the SM-associated locus on CFA26 and pain in our cohort of 65 CKCS and we did not detect any significant association. However, at the time of DNA collection, phenotyping for pain was not as rigorous and pain relating to CM versus SM pain was not separated. Moreover, objective phenotyping for pain, a subjective experience, is extremely difficult especially when it is partly dependent on owner reporting. Genetic investigation in larger CKCS cohorts with improved pain phenotyping data is needed to further analyze the role of *ZWINT* in pain development and in SM associated with CM.

CM with SM in the dog is very similar to a condition in humans called Chiari malformation I (CMI) with a reported frequency of 1 in 1280. As in dogs, the prevalence of SM secondary to CMI in humans is high reaching 65%–85% [47, 48]. Genetic studies of CM and SM in both humans and dogs clearly suggest a complex genetic architecture which has hampered the identification of predisposing genetic factors. The dog model is the only known naturally-occurring animal model for CMI in humans. The reduced genetic variability of dogs caused by founder effects, genetic bottlenecks and strong inbreeding make it an excellent tool for investigation of complex diseases [49] . Hence, gene identification studies in CM with SM in the dog might provide an entry point for identification of novel genes and pathways involved in the pathogenesis of CMI and SM in humans.

## Conclusions

In this study, we have used a genome-wide association study to decipher the genetics of SM secondary to CM in the CKCS breed. We identified 6 cranial T1-weighed sagittal MRI measurements that were associated with the syrinx transverse diameter. We next identified 2 haplotypes on CFA22 and CFA26 that were significantly associated to ratio F-d/BC and *L4 + L7* respectively. Genotyping of a larger cohort of CKCS dogs confirmed association of the locus on CFA22 with SM in this breed. Each of these 2 haplotypes harbored only one gene: *PCDH17* on CFA22 that codes for a cell adhesion molecule specifically expressed in the brain and spinal cord and *ZWINT* that plays a role in proper chromosome segregation and whose expression is increased with the onset of neuropathic pain. Additional molecular genetic studies in larger CKCS cohorts from various affected brachycephalic breeds and in cell and animal models are needed to further investigate the role of the 2 associated loci and the genes they harbor in the pathogenesis of SM secondary to CM. Our study represents an essential step towards a better understanding of the complex genetics of this devastating condition and development of breeding strategies that aim at eliminating it from the affected dog breeds. It also provides an important model for studying CMI/SM in humans.

## Additional files

Additional file 1: **Table S1.** Characteristics and measurements of 96 CKCS of the cohort. This table includes the gender, age, clinical status and all MRI cranial measurements taken on the 96 CKCS dogs included in this study. (XLSX 41 kb)

Additional file 2: **Table S2.** SNPs suggestive of association to SM in the CKCS breed. This table enlists all SNPs suggestive of association with FDR corrected scores between 0.05 and 0.1. These SNPs were identified following a GWAS using a mixed linear model with age as a covariate on the previously identified traits (Line AE, line AI, angle 3, angle 7, ratio F-d/BC and *L*4 + *L*7). (DOCX 15 kb)

Additional file 3: **Figure S1.** *P* value distribution inside the CFA15 (24537882-26,252,411 bp) associated region. This region spans 1.7 Mb surrounding SNPs associated to ratio F-d/BC and was identified using Haploview V4.2. (PDF 54 kb)

## Abbreviations

CFA: *Canis familiaris* autosome
CM: Chiari-like malformation
CSF: Cerebrospinal fluid
DICOM: Digital Imaging and Communications in Medicine
GB: Griffon Bruxellois
GWAS: Genome-wide association study
Mb: Mega basepairs
MRI: Magnetic Resonance Imaging
PCDH17: Protocadherin 17
SM: Syringomyelia
STD: Syrinx transverse diameter
TagSNPs: Tagging single nucleotide polymorphisms
ZWINT: ZW10-interactor
