# Supplementary material for: A genome-wide association study identifies candidate loci associated to syringomyelia secondary to Chiari-like malformation in Cavalier King Charles Spaniels
Source: BMC Genet. 2018 Mar 22;19:16. doi: 10.1186/s12863-018-0605-z (PMC5865342; doi:10.1186/s12863-018-0605-z)
Supplement: Supplementary file 2 — Table S2. SNPs suggestive of association to SM in the CKCS breed. This table enlists all SNPs suggestive of association with FDR corrected scores between 0.05 and 0.1. These SNPs were identified following a GWAS using a mixed linear model with age as a covariate on the previously identified traits (Line AE, line AI, angle 3, angle 7, ratio F-d/BC and L4 + L7). (DOCX 15 kb) [file 12863_2018_605_MOESM2_ESM.docx]

| Table S2. SNPs suggestive of association to SM in the CKCS breed | | | | | |
| --- | --- | --- | --- | --- | --- |
| Chr | SNP | Position | *P* value | *Q* value | Trait |
| 5 | BICF2P694044 | 9144363 | 6.76E-06 | 0.097777 | FDBC |
| 5 | BICF2P754020 | 10259031 | 4.10E-06 | 0.069782 | FDBC |
| 11 | BICF2P1267841 | 75010057 | 2.97E-06 | 0.069782 | AE |
| 12 | TIGRP2P165985_rs9097044 | 49228547 | 2.61E-06 | 0.069782 | AI |
| 15 | BICF2S22961368 | 26586223 | 1.11E-06 | 0.037545 | FDBC |
| 15 | BICF2G630437186 | 26599059 | 1.11E-06 | 0.037545 | FDBC |
| 15 | BICF2G630437178 | 26605637 | 1.11E-06 | 0.037545 | FDBC |
| 15 | BICF2G630437135 | 26619845 | 1.11E-06 | 0.037545 | FDBC |
| 15 | BICF2G630437112 | 26623178 | 1.11E-06 | 0.037545 | FDBC |
| 15 | BICF2G630437075 | 26645302 | 1.11E-06 | 0.037545 | FDBC |
| 15 | BICF2G630437073 | 26645969 | 1.11E-06 | 0.037545 | FDBC |
| 15 | BICF2S23311892 | 26690382 | 1.11E-06 | 0.037545 | FDBC |
| 15 | BICF2G630437043 | 26734763 | 1.11E-06 | 0.037545 | FDBC |
| 15 | BICF2G630437038 | 26738248 | 1.11E-06 | 0.037545 | FDBC |
| 15 | BICF2G630437002 | 26797343 | 1.11E-06 | 0.037545 | FDBC |
| 15 | BICF2G630436158 | 28046943 | 4.28E-06 | 0.069782 | FDBC |
| 15 | BICF2S23534119 | 28416735 | 4.28E-06 | 0.069782 | FDBC |
| 15 | BICF2S23761321 | 28798671 | 3.81E-07 | 0.037545 | FDBC |
| 15 | G881f57S156 | 28860127 | 4.28E-06 | 0.069782 | FDBC |
| 15 | BICF2P615863 | 28937930 | 4.28E-06 | 0.069782 | FDBC |
| 15 | BICF2G630435532 | 28947608 | 4.28E-06 | 0.069782 | FDBC |
| 15 | BICF2G630435512 | 28980844 | 4.28E-06 | 0.069782 | FDBC |
| 15 | BICF2G630435496 | 28996713 | 4.28E-06 | 0.069782 | FDBC |
| 15 | BICF2G630435491 | 29013019 | 4.28E-06 | 0.069782 | FDBC |
| 15 | BICF2G630435471 | 29027536 | 3.35E-06 | 0.069782 | FDBC |
| 15 | BICF2G630435442 | 29066852 | 4.28E-06 | 0.069782 | FDBC |
| 15 | BICF2G630435384 | 29131922 | 4.28E-06 | 0.069782 | FDBC |
| 15 | BICF2G630435380 | 29147043 | 5.87E-07 | 0.037545 | FDBC |
| 19 | BICF2P910806 | 4820716 | 4.82E-06 | 0.07615 | AI |
| 22 | BICF2P1045632 | 17095566 | 5.20E-06 | 0.078464 | FDBC |
| 26 | BICF2S2335627 | 35463330 | 1.96E-06 | 0.060292 | L4L7 |
| 26 | BICF2P174010 | 35796851 | 5.99E-07 | 0.037545 | L4L7 |
| 26 | BICF2P152116 | 35799961 | 5.99E-07 | 0.037545 | L4L7 |
| 26 | BICF2P518550 | 35988365 | 5.27E-06 | 0.078464 | L4L7 |
| 26 | BICF2S23544425 | 36132126 | 2.03E-06 | 0.060292 | L4L7 |
