# Supplementary figures and images for: A genome-wide association study identifies candidate loci associated to syringomyelia secondary to Chiari-like malformation in Cavalier King Charles Spaniels
Source: BMC Genet. 2018 Mar 22;19:16. doi: 10.1186/s12863-018-0605-z (PMC5865342; doi:10.1186/s12863-018-0605-z)

CFA15

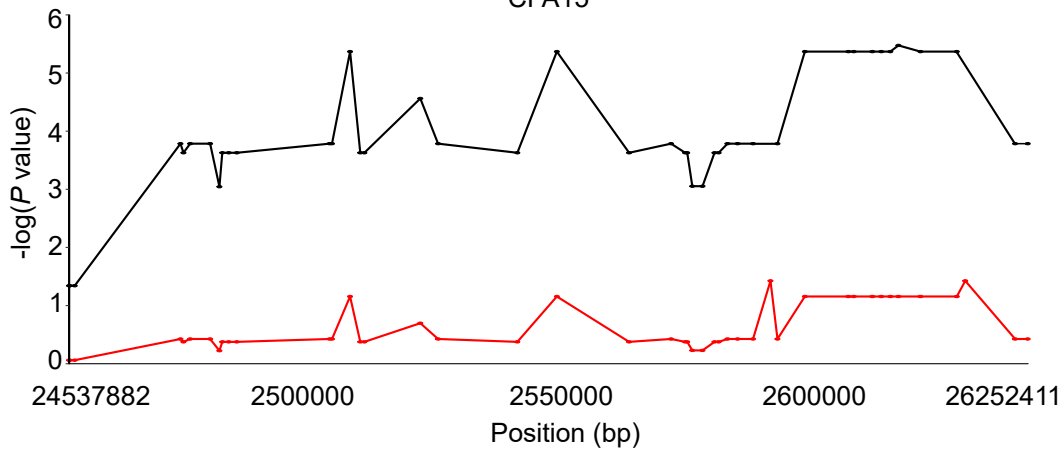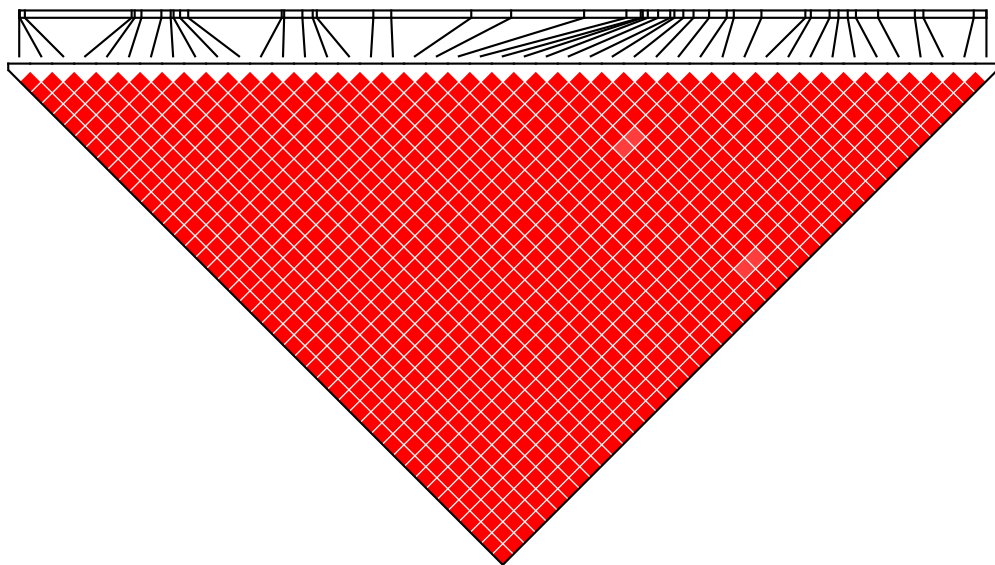

Supplement: Supplementary file 3 — Figure S1. P value distribution inside the CFA15 (24537882-26,252,411 bp) associated region. This region spans 1.7 Mb surrounding SNPs associated to ratio F-d/BC and was identified using Haploview V4.2. (PDF 54 kb) [file 12863_2018_605_MOESM3_ESM.pdf]
